# Prognostic Significance of Inflammatory Biomarkers in First-Line Immunotherapy for Metastatic Melanoma: Multicentric Study †

**DOI:** 10.3390/cancers18111722

**Published:** 2026-05-25

**Authors:** Branko Dujovic, Aleksandar Popovic, Amina Jalovcic Suljevic, Bojana Cikota-Aleksic, Mirjana Balic, Igor Salatic, Jovana Pavlica, Philipp Schnecko, Tanja Mesti, Muamer Terzo, Emina Bicakcic Filipovic, Lidija Kandolf

**Affiliations:** 1 Clinic for Dermatovenerology, Military Medical Academy, University of Defense, 11000 Belgrade, Serbia; 2 Faculty of Medicine, Military Medical Academy, University of Defense, 11000 Belgrade, Serbia; 3 Clinic for Oncology, Clinical Centre of Niš, 18000 Niš, Serbia; 4 Clinic of Oncology, Clinical Center University of Sarajevo, 71000 Sarajevo, Bosnia and Herzegovina; 5 Centre for Clinical Pharmacology, Military Medical Academy, 11000 Belgrade, Serbia; 6 Alcedis, a HUMA Company, 35394 Giessen, Germany; 7 Institute of Oncology, 1000 Ljubljana, Slovenia

**Keywords:** metastatic melanoma, first-line immunotherapy, immune checkpoint inhibitors, inflammatory biomarkers, progression-free survival, overall survival, prognostic factors

## Abstract

**Background/Objectives**: This study evaluates the prognostic value of baseline inflammatory biomarkers, neutrophil-to-lymphocyte ratio (NLR), platelet-to-lymphocyte ratio (PLR), monocyte-to-lymphocyte ratio (MLR), lymphocyte-to-monocyte ratio (LMR), systemic immune-inflammation index (SII), and pan-immune-inflammation value (PIV) in advanced cutaneous melanoma treated with first-line immunotherapy. **Methods**: This multicenter retrospective study included 162 patients with unresectable stage III/IV cutaneous melanoma treated with first-line pembrolizumab, nivolumab, or nivolumab plus ipilimumab. Biomarkers were calculated from complete blood counts obtained within 30 days before treatment start. Cut-offs were defined by ROC analysis. Progression-free survival (PFS) and overall survival (OS) were analyzed using Kaplan–Meier and Cox regression. Response was assessed by RECIST v1.1. **Results**: Higher baseline NLR, PLR, MLR, SII, and PIV were more common in patients with adverse baseline features, including liver metastases, elevated LDH, and poorer ECOG performance status. Patients with biomarker values below the cut-offs had significantly longer PFS and OS. In biomarker-specific multivariable models adjusted for selected clinical covariates, PIV retained the most consistent association with PFS and OS, while MLR was associated with PFS, and PLR with OS. **Conclusions**: Baseline inflammatory biomarkers from routine blood counts provide useful prognostic information in advanced melanoma treated with first-line ICIs. PIV showed the most consistent association with survival outcomes and may support initial risk stratification alongside LDH, ECOG, and metastasis pattern. However, prospective validation in independent cohorts is needed before routine clinical implementation.

## Simple Summary

Patients with advanced melanoma treated with first-line immunotherapy do not all have the same outcome, and simple tools to identify higher-risk patients at treatment start are still needed. In this multicenter retrospective study, we evaluated whether inflammatory biomarkers calculated from routine blood counts before treatment were associated with survival outcomes in 162 patients with unresectable stage III or IV cutaneous melanoma. Patients with less favorable biomarker profiles had shorter progression-free and overall survival. Among the evaluated markers, the pan-immune-inflammation value showed the most consistent association with survival outcomes after adjustment for selected clinical factors. These findings suggest that inexpensive and widely available blood-based inflammatory markers may help improve initial risk stratification in advanced melanoma, alongside established clinical factors. However, prospective validation in independent cohorts is required before these markers can be used routinely in clinical practice.

## 1. Introduction

Melanoma is a significant global health concern, with rising incidence and mortality rates in recent years [1]. The main predictor for survival is tumor stage at diagnosis, ranging from 85% to 95% at 10 years for localized disease, while survival rates for advanced melanoma are much lower [2]. Cutaneous melanoma develops through complex interactions between environmental exposure, host factors, tumor genetics, and immune regulation. Ultraviolet radiation is a major environmental driver of melanomagenesis, inducing DNA damage, oxidative stress, impaired DNA repair, and inflammatory responses within the skin microenvironment [3]. These processes contribute to melanoma initiation and progression and provide a broader biological context for understanding the interaction between tumor biology and host immune-inflammatory responses. In advanced disease, systemic inflammatory indices derived from peripheral blood may reflect, at least in part, the balance between tumor burden, host immune competence, and cancer-related inflammation [4,5].

Immunotherapy has reshaped the management of metastatic disease. Anti–PD-1 monotherapy with pembrolizumab or nivolumab yields durable benefit, with five-year survival rates of 38.7% and 39%, respectively [6,7]. Combination of nivolumab and ipilimumab provides additional advantage, achieving five-year overall survival of up to 52%^5^. These data underscore the central role of immunotherapy in prolonging survival in advanced melanoma. Although immunotherapy improves outcomes in metastatic melanoma, primary resistance is still common. Up to 58% of patients receiving anti-PD-1 monotherapy and up to 42% receiving anti–PD-1 plus anti–CTLA-4 do not achieve an objective response in the first-line setting [7,8,9]. In view of persistent primary and acquired resistance, priority should be given to individualizing management and to early identification of patients who will benefit from first-line immunotherapy.

So far, some tumor-related factors have been suggested as predictive markers of efficacy for immunotherapy [10,11,12]. In routine practice, however, rapid and low-cost biomarkers are needed to support treatment individualization [13,14]. Growing evidence implies that systemic inflammatory response impacts disease progression and course in different cancers, including melanoma [4,14]. Hence, several immune-based scores such as neutrophil count, lymphocyte count, neutrophil-to-lymphocyte ratio (NLR), platelet-to-lymphocyte ratio (PLR), and monocyte-to-lymphocyte ratio (MLR) were usually employed to assess the systemic inflammation in cancer patients [5,13,15,16]. Low lymphocyte count, increased neutrophil or platelet count are frequently observed in cancer patients, indicating poor survival outcome [14,15,16,17,18]. These counts can be used for the calculation of pan-immune-inflammation value (PIV), a new comprehensive biomarker that has been proven to be a strong predictor of survival in patients with metastatic colorectal cancer, non-small cell lung cancer, and breast cancer [19,20,21].

Therefore, there is a need to further evaluate whether routinely available blood-based inflammatory biomarkers can provide clinically meaningful prognostic information in patients with advanced melanoma treated with first-line immune checkpoint inhibitors. In this multicenter retrospective study, we assessed the prognostic value of baseline NLR, PLR, MLR, LMR, SII, and PIV in patients with unresectable stage III or IV cutaneous melanoma receiving first-line pembrolizumab, nivolumab, or nivolumab plus ipilimumab. We evaluated the association of these biomarkers with baseline clinicopathological characteristics, response to therapy, progression-free survival, and overall survival. By comparing several inflammatory indices within the same real-world cohort, this study aimed to identify which markers may provide the most consistent prognostic information and to clarify their potential role in initial risk stratification. Given the retrospective design and the exploratory nature of the cut-off values, the findings are intended to support further validation rather than immediate clinical implementation.

## 2. Materials and Methods

### 2.1. Patients

The study enrolled 162 patients with advanced cutaneous melanoma treated at three centers in the Western Balkans (South Eastern Europe)—two in Serbia (the Clinic of Dermatolovenereology, Military Medical Academy, Belgrade [*n* = 68] and the Clinic of Oncology, University Clinical Center Niš [*n* = 88]) and one in Bosnia and Herzegovina (the Clinic of Oncology, Clinical Center Sarajevo [*n* = 6]). Inclusion criteria for participation in the study were: 18 years of age or older, unresectable stage III/IV cutaneous melanoma, first-line mono-immunotherapy (pembrolizumab or nivolumab) or combined immunotherapy (nivolumab and ipilimumab), and availability of a complete blood count with differential obtained within 30 days before initiation of the first treatment cycle. Exclusion criteria were: age under 18 years, patients with insufficient follow-up (<12 months) without documented progression or death.

### 2.2. Treatment

In the pembrolizumab regimen, 200 mg of pembrolizumab was administered intravenously every 3 weeks or 400 mg every 6 weeks. In the nivolumab regimen, 480 mg of nivolumab was administered intravenously every 4 weeks. In the ipilimumab plus nivolumab regimen, 3 mg/kg of ipilimumab was administered intravenously following 1 mg/kg of nivolumab, or 1 mg/kg of ipilimumab following 3 mg/kg of nivolumab, every 3 weeks for up to 4 cycles, followed by continued administration of 480 mg of nivolumab every 4 weeks. Dose and schedule modifications were permitted based on the patient’s condition.

Response to therapy was evaluated according to Response Evaluation Criteria in Solid Tumors (RECIST v1.1) guidelines [22].

### 2.3. Data Collection and Ethics

Patients’ data were extracted from the National Melanoma Registries, which are part of the Central South Eastern European Melanoma Expert Group, and integrated into the European Melanoma Registry (EuMelaReg).

The collection of patient data and its entry into the National Melanoma Registries were approved by the Ethics Committees of the respective institutions from which the data were obtained (Ethics Committee of the Military Medical Academy, Serbia, Number 55/2019 from 4 July 2019; Ethics Committee of the Clinical Center Niš, Serbia, Number 13872/502 from 6 June 2020; Ethics Committee of the University Clinical Center Sarajevo, Bosnia and Herzegovina, Number 03-02-282 from 24 December 2019).

All participants had previously signed informed consent forms allowing their clinicopathological data to be entered into an anonymized melanoma registry and used for research purposes.

### 2.4. Statistical Analysis

The baseline clinicopathological variables were summarized using descriptive statistics.

Calculation of biomarker ratios included absolute cell counts at baseline (within 30 days before the initiation of immunotherapy). PIV was calculated as (neutrophil count × platelet count × monocyte count)/lymphocyte count, while SII was calculated as (neutrophil count × platelet count)/lymphocyte count. All baseline absolute cell counts were in 10^9^/L.

The cut-off values were calculated using ROC-Curves predicting a survival of “>1 year”, choosing the value with the highest distance from the diagonal.

Progression-free survival (PFS) was calculated from treatment initiation to radiologically confirmed disease progression according to RECIST criteria or death from any cause. Participants who started a new therapy before progression/death were censored at the start of the next therapy. Participants without progression/death and no further therapies were censored at the last date they were known to be alive. Overall survival (OS) was calculated from the start of treatment until death. Participants not documented as dead were censored at the last date they were known to be alive. Survival curves were plotted using the Kaplan–Meier method.

Univariable Cox proportional hazards regression was used to evaluate associations between baseline inflammatory biomarkers, clinicopathological variables, and survival outcomes. Biomarker-specific multivariable Cox proportional hazards models were then fitted separately for each inflammatory biomarker and adjusted for selected clinically relevant covariates. Because several inflammatory indices share the same cellular components, biomarkers were evaluated in separate biomarker-specific multivariable Cox models rather than simultaneously in a single model, to reduce potential multicollinearity. Hazard ratios (HRs) with 95% confidence intervals (CIs) were reported.

*p* values were calculated using Fisher’s exact test for categorical variables, Student’s *t*-test for continuous variables, and Log-Rank-Test for Kaplan–Meier-estimates.

Missing data are presented, but not imputed. *p* values less than 0.05 were considered statistically significant.

## 3. Results

### 3.1. Clinical Characteristics and Therapy

This study included 61 females (37.65%) and 101 men (62.35%) with advanced cutaneous melanoma. The mean age at initiation of immunotherapy was 65 years (range 28–96, median 67). The most frequent histological subtype was nodular melanoma (54.94%). Median Breslow thickness was 4.45 mm (range 0.4–38 mm), and ulceration was present in 108 patients (66.67%), indicating that more than half of the cohort initially had histopathologically high-risk cutaneous melanoma. BRAF mutation was detected in 63 patients (38.89%). BRAF V600 mutation status was assessed on formalin-fixed paraffin-embedded tumor tissue using real-time PCR-based assays according to institutional protocols. Testing was performed as part of routine clinical molecular diagnostics at the participating institutions. At initial diagnosis, most patients were stage II (55.56%), whereas 7.4% were already stage IV. The mean interval from initial diagnosis to relapse to stage III or IV was 28.1 months (range 0–165.16; median 17.01). Table 1 presents clinicopathologic characteristics at baseline.

As the first treatment, 59.88% received pembrolizumab, 36.42% received nivolumab, and 3.70% received combination nivolumab/ipilimumab in standard regimen. At the start of immunotherapy, most patients had involvement of two or fewer metastatic organ sites (69.75%). The most common sites of metastasis were the lymph nodes (55.56%), lungs (50.62%), skin/subcutaneous (31.48%), and liver (27.16%). Central nervous system metastases were present in 17 patients (10.49%). Baseline LDH values were elevated in 57 patients (35.19%). Most patients had an ECOG performance status of 0 or 1 (93.83%). Best overall response to first-line therapy comprised a complete response (CR) in 15.43% of patients; the disease control rate (DCR) was 66.05%, and the objective response rate (ORR) 37.04%. Immune-related adverse events were documented in 58 patients.

### 3.2. ROC Analyses and Cut-Off Values of Inflammatory Biomarkers

The ROC curves were calculated against OS > 1 year vs. OS ≤ 1 year. Baseline cut-offs for inflammatory biomarkers were: NLR 3.748 [area under the curve (AUC) 0.66; *p* = 0.012], PLR 180.741 (AUC 0.614; *p* = 0.003), MLR 0.298 (AUC 0.658; *p* = 0.005), LMR 3.351 (AUC 0.652; *p* = 0.083), SII 763.958 (AUC 0.623; *p* = 0.001), and PIV 277.269 (AUC 0.62; *p* = 0.003).

### 3.3. Inflammatory Biomarkers and Clinical Characteristics

Patients with baseline NLR, PLR, and MLR values above the cut-off more frequently experienced liver metastases (*p* = 0.002 for NLR; *p* = 0.004 for PLR; *p* = 0.043 for MLR), and elevated serum LDH (*p* = 0.001 for NLR; *p* < 0.0001 for PLR; *p* = 0.001 for MLR). These patients were also older at the beginning of immunotherapy (*p* = 0.043 for NLR; *p* = 0.015 for PLR; *p* = 0.002 for MLR), and had unfavorable ECOG score (*p* = 0.001 for NLR; *p* = 0.004 for PLR; *p* = 0.004 for MLR) and lower rate of overall response (ORR) to therapy (*p* = 0.004 for NLR; *p* < 0.0001 for PLR; *p* = 0.028 for MLR), respectively. Microsatellites were more frequently present in patients with NLR and PLR values above the cut-off than in patients with lower NLR and PLR (*p* = 0.055 and *p* = 0.065). PLR above threshold was also associated with the presence of more than two metastatic sites (*p* = 0.039), while MLR values above threshold were also associated with elevated serum S-100 (*p* = 0.002).

When patients were grouped by at least one high ratio (NLR, PLR or MLR), significant association was observed for older age at the start of immunotherapy (*p* = 0.005), presence of liver metastasis (*p* = 0.033), elevated serum LDH (*p* = 0.0001) and S-100 (*p* = 0.039), unfavorable EGOG score (*p* = 0.003) and ORR (*p* = 0.006).

Analysis of LMR showed that patients with values above the cut-off were younger at the start of immunotherapy (median 64 vs. 70.5 years, *p* = 0.004), less frequently had metastases in the liver (21.35% vs. 35.94%, *p* = 0.066), elevated serum LDH (25.84% vs. 45.32%, *p* = 0.002), and S-100 (3.37% vs. 20.31%, *p* = 0.003). Also, these patients less frequently had poor ECOG score (24.72% vs. 40.63%, *p* = 0.051) and more frequently had better ORR (42.7% vs. 25%, *p* = 0.027).

Patients with baseline SII above the cut-off more frequently had liver metastases (*p* = 0.0034), more than two metastatic sites (*p* = 0.053), elevated serum LDH (*p* = 0.0002) and S-100 protein (*p* = 0.051). These patients were also older at the start of immunotherapy (*p* = 0.031), and more frequently had unfavorable ECOG scores (*p* = 0.002) and poor ORR to therapy (*p* = 0.007).

PIVs above the cut-off were associated with elevated LDH (*p* < 0.0001) and S-100 (*p* = 0.01), unfavorable ECOG score (*p* = 0.022), and poor ORR (*p* = 0.042).

Data on the significant association between inflammatory biomarkers and clinical characteristics are presented in Table 2.

### 3.4. Inflammatory Biomarkers and Survival Analyses

Patients with baseline values below these thresholds had markedly longer median PFS: 11.48 vs. 3.06 months for NLR (*p* < 0.0001); 11.48 vs. 3.95 months for PLR (*p* = 0.0006); 11.18 vs. 4.93 months for MLR (*p* < 0.0001); 13.91 vs. 3.85 months for PIV (*p* < 0.0001). The same pattern was observed for OS, with significantly longer survival in the below cut-off groups: 29.18 vs. 5.03 months for NLR (*p* < 0.0001); 29.87 vs. 6.78 months for PLR (*p* < 0.0001); 49.90 vs. 8.42 months for MLR (*p* < 0.0001); 33.09 vs. 8.42 months for PIV (*p* < 0.0001).

Using a composite indicator, patients were classified as having at least one high biomarker if at least one of NLR, PLR, or MLR exceeded its pre-specified cut-off (with the other two below). Compared with the all-low group, at least one high cohort had markedly shorter survival—median PFS 4.21 vs. 16.02 months and OS 8.42 vs. 52.66 months, both *p* < 0.0001.

SII values below the cut-off were associated with superior median PFS (13.91 vs. 4.21 months, *p* = 0.0083) and OS (29.18 vs. 7.4 months, *p* = 0.0007).

Opposite to NLR, MLR, PLR, and SII patients with LMR values below the threshold, had poor median PFS (5 vs. 11.18 months, *p* < 0.0001) and OS (8.42 vs. 49.9 months, *p* < 0.0001).

**Figure 1:**
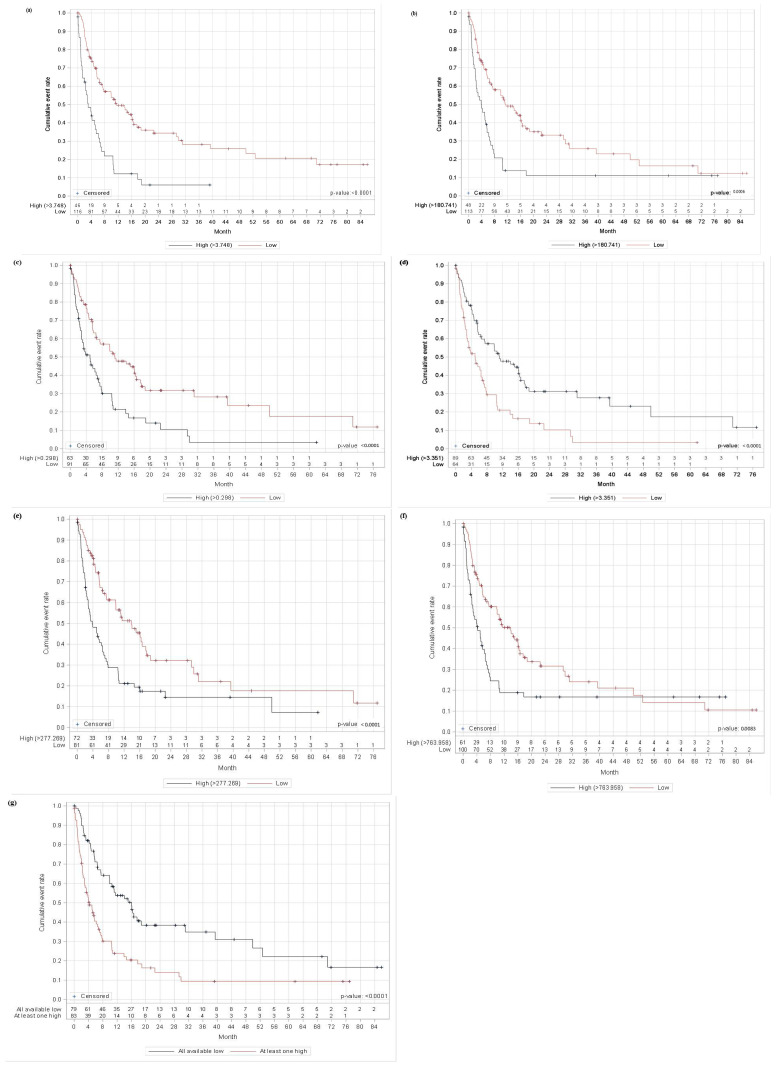
Kaplan–Meier curves for progression-free survival (PFS) according to baseline inflammatory biomarkers. PFS, progression-free survival; NLR, neutrophil-to-lymphocyte ratio; PLR, platelet-to-lymphocyte ratio; MLR, monocyte-to-lymphocyte ratio; LMR, lymphocyte-to-monocyte ratio; SII, systemic immune-inflammation index; PIV, pan-immune-inflammation value. ROC-derived cut-offs and *p*-values: (**a**) NLR (3.748; *p* < 0.0001), (**b**) PLR (180.741; *p* = 0.0006), (**c**) MLR (0.298; *p* < 0.0001), (**d**) LMR (3.351; *p* < 0.0001), (**e**) PIV (277.969; *p* < 0.0001), (**f**) SII (763.958; *p* = 0.0083), and (**g**) all available biomarkers low vs. at least one high (*p* < 0.0001).

**Figure 2:**
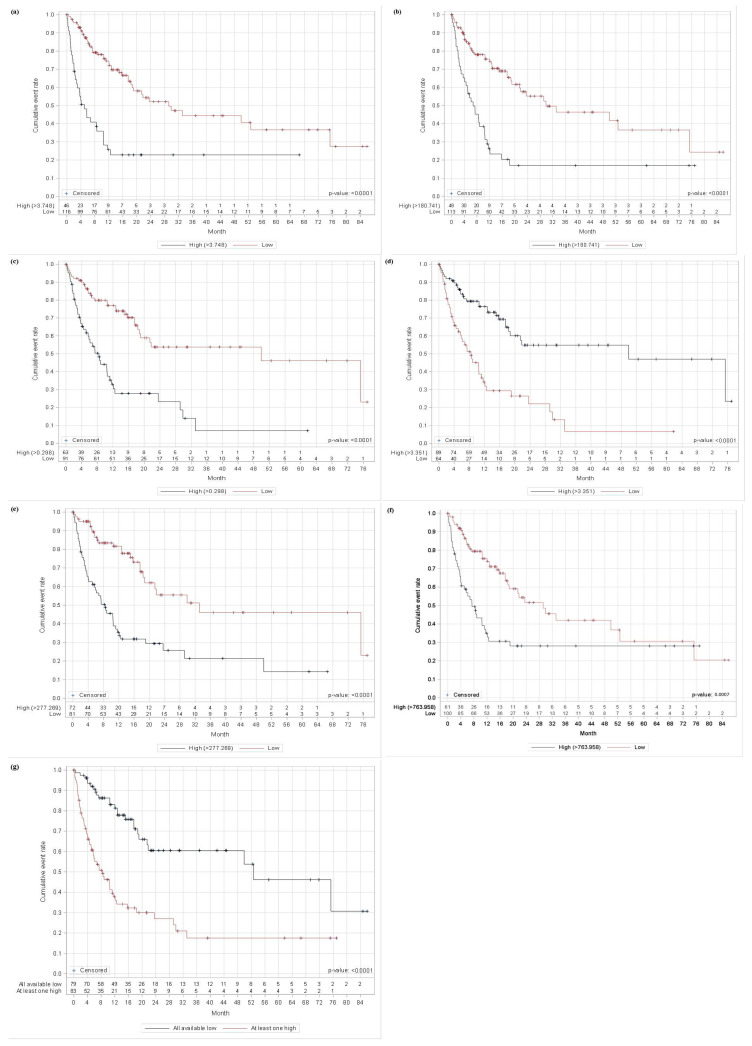
Kaplan–Meier curves for overall survival (OS) according to baseline inflammatory biomarkers. OS, overall survival; NLR, neutrophil-to-lymphocyte ratio; PLR, platelet-to-lymphocyte ratio; MLR, monocyte-to-lymphocyte ratio; LMR, lymphocyte-to-monocyte ratio; SII, systemic immune-inflammation index; PIV, pan-immune-inflammation value. ROC-derived cut-offs and *p*-values: (**a**) NLR (3.748; *p* < 0.0001), (**b**) PLR (180.741; *p* < 0.0001), (**c**) MLR (0.298; *p* < 0.0001), (**d**) LMR (3.351; *p* < 0.0001), (**e**) PIV (277.969; *p* < 0.0001), (**f**) SII (763.958; *p* = 0.0007), and (**g**) all available biomarkers low vs. at least one high (*p* < 0.0001).

Survival curves are presented in Figure 1 and Figure 2.

### 3.5. Cox Regression Analysis

Univariate Cox regression for PFS and OS, respectively, included the following variables: NLR, PLR, MLR, LMR, SII, PIV, gender, age, histologic subtype, Breslow thickness, the presence of ulcerations, lymphovascular and/or perineural invasion, tumor infiltrating lymphocytes, microsatellites before the first line of therapy, BRAF status, number of metastatic sites, the presence of CNS and/or liver and/or lung metastases, clinical stage, baseline levels of LDH and S100, ECOG score and prior adjuvant treatment.

In univariate Cox analysis, higher baseline NLR (*p* = 0.006), PLR (*p* = 0.001), MLR (*p* < 0.0001), PIV (*p* < 0.0001) and SII (*p* < 0.0001), as well as the presence of acral lentiginous melanoma (*p* = 0.003), LDH > 2× Upper Limit of Normal (ULN) (*p* = 0.0002) and ECOG score > 0 (*p* = 0.002) were associated with shorter PFS. Opposite, higher baseline LMR (*p* = 0.015) and the absence of perineural invasion (*p* = 0.011) indicate better PFS.

For OS, all five indices were likewise significant: NLR (*p* = 0.002), PLR (*p* < 0.0001), MLR (*p* < 0.0001), PIV (*p* < 0.0001), and SII (*p* < 0.0001). Inferior OS was also associated with age over 60 years (*p* = 0.016), the presence of nodular (*p* = 0.028) or acral lentiginous melanoma (*p* = 0.0001), wild-type BRAF (*p* = 0.019), baseline LDH elevated (*p* = 0.004) or LDH > 2× ULN (*p* < 0.0001), and ECOG score > 0 (*p* < 0.0001). Similarly to PFS, LMR above cut-off values (*p* = 0.002) and the absence of perineural invasion (*p* = 0.009) indicate better OS. In multivariable Cox models fitted separately for each biomarker and adjusted for clinical covariates, MLR (*p* = 0.006) and PIV (*p* = 0.001) retained independent associations with shorter PFS. For OS, PLR (*p* = 0.032) and PIV (*p* = 0.032) remained independently associated with mortality, whereas NLR and MLR were not significant after adjustment. In these adjusted models, additional covariates showed consistent associations: ECOG performance status >0 predicted shorter PFS in the NLR (*p* = 0.013), PLR (*p* = 0.02), and MLR (*p* = 0.016) models. Baseline LDH >2× ULN predicted worse PFS in the NLR (*p* = 0.002) and PLR (*p* = 0.011) models, and worse OS in the NLR (*p* < 0.0001), PLR (*p* = 0.001), and MLR (*p* = 0.004) models. For OS, older age was independently associated with higher mortality in the PLR (*p* = 0.009), MLR (*p* = 0.01), and PIV (*p* = 0.002) models. The acral lentiginous subtype conferred higher risk for PFS in the NLR (*p* = 0.043), MLR (*p* = 0.02) and PIV (*p* = 0.01) models, and for OS in the PLR (*p* = 0.023), MLR (*p* = 0.01) and PIV (*p* = 0.013) models. Finally, the absence of CNS metastases was associated with better OS in the PLR (*p* = 0.0392), MLR (*p* = 0.024), and PIV (*p* = 0.012) models. The most impactful variables of the multivariable Cox proportional-hazards analyses for PFS and OS are summarized in Table 3.

Results of all biomarker-specific univariable and multivariable Cox regression models for PFS and OS are provided in Supplementary Tables S1 and S2.

## 4. Discussion

In this multicenter, real-world cohort of patients with unresectable stage III/IV cutaneous melanoma receiving first-line immune checkpoint inhibitors (ICIs), baseline inflammatory biomarkers derived from routine complete blood counts showed clinically meaningful and partly independent prognostic associations. In univariable analyses, higher NLR, PLR, monocyte–lymphocyte ratio, and PIV were each linked to shorter PFS and OS. After adjustment for clinical covariates in the biomarker-specific multivariable Cox models, the monocyte–lymphocyte ratio and PIV retained independent values for PFS, while PLR and PIV remained independently associated with OS. These findings support the concept that composite complete blood count–derived markers reflecting the balance between innate (neutrophils, monocytes, platelets) and adaptive (lymphocytes) immunity have been investigated as baseline tools for risk stratification in ICI-treated melanoma, although results across cohorts are not fully consistent [5,23,24,25]. Nevertheless, the adjusted associations should be interpreted with caution. Several inflammatory biomarkers evaluated in this study share overlapping cellular components; for example, neutrophils, lymphocytes, and platelets contribute to multiple indices, while PIV integrates neutrophils, platelets, monocytes, and lymphocytes into a single composite score. This overlap may lead to multicollinearity and may partly explain why some biomarkers lost statistical significance after adjustment, whereas others retained only borderline associations.

Our results are consistent with a meta-analysis of melanoma cohorts treated with ICIs (*n* = 3235), in which elevated baseline NLR and PLR were associated with poorer OS and PFS, supporting the prognostic utility of systemic inflammatory biomarkers at treatment initiation [5].

For PIV, published data on melanoma are not fully concordant. In a retrospective single-center cohort of metastatic melanoma receiving first-line systemic therapy (including both immunotherapy and targeted therapy), higher baseline PIV was independently associated with shorter PFS and OS, whereas a smaller single-center study restricted to ICI-treated advanced melanoma did not confirm significant associations with response or survival outcomes. A recent meta-analysis across ICI-treated cancers further supported the adverse prognostic impact of elevated PIV on both PFS and OS [23,24,25].

The observed link between higher inflammatory biomarkers and worse baseline features (higher LDH, poorer ECOG performance status, and visceral disease) is biologically and clinically plausible. LDH is widely used as a marker of tumor burden, but it also reflects a more glycolytic tumor environment with higher lactate production, which can impair effector T-cell function and contribute to weaker antitumor immunity [26,27]. Regarding liver involvement, we interpret it as a feature of high-risk visceral spread rather than a single isolated factor; importantly, a recent multicenter nomogram in unresectable stage IV melanoma treated with first-line anti-PD-1–based therapy included the presence of liver or brain metastases together with LDH, NLR, melanoma subtype, and other variables as key determinants of early progression risk [28]. This approach is consistent with current ESMO guidance, which stresses baseline clinical assessment—including LDH, performance status, and the pattern of visceral disease—when choosing and sequencing systemic treatment in advanced melanoma [29].

From a biological perspective, CBC-derived inflammatory markers may provide an indirect snapshot of the balance between protumor innate inflammation and adaptive antitumor immunity. Neutrophils can support tumor progression through inflammatory mediators, reactive oxygen species, proteases, and neutrophil extracellular traps (NETs), which may facilitate tumor invasion, metastatic dissemination, immune evasion, and resistance to anticancer therapies, including immunotherapy [30]. Monocytes may also contribute to tumor-promoting inflammation through recruitment into the tumor microenvironment and differentiation toward tumor-associated macrophage or myeloid-derived suppressor cell-like populations, which can suppress cytotoxic T-cell activity and support immune escape [31]. Platelets are active mediators of cancer progression; they can protect circulating tumor cells from immune recognition, promote angiogenesis and vascular remodeling, and contribute to metastatic niche formation [32,33,34,35]. In contrast, lymphocytes are central mediators of adaptive antitumor immunity and represent the immune compartment most directly reinvigorated by immune checkpoint blockade. Accordingly, lower circulating lymphocyte levels and reduced tumor-infiltrating lymphocyte activity have been associated with poorer outcomes in melanoma and other ICI-treated cancers [36,37]. Taken together, indices that combine increased neutrophil, monocyte, or platelet counts with reduced lymphocyte counts—such as NLR, PLR, MLR, SII, and particularly PIV—may reflect a systemic immune-inflammatory state that is less favorable for effective ICI-mediated antitumor responses [5,23,24,25,30,31,32,33,34,35,36,37]. However, this interpretation remains biologically plausible but indirect, because our retrospective study did not include functional immune profiling or tumor microenvironment analyses.

Clinically, these markers are attractive because they are inexpensive, readily available, and reproducible. In our cohort, PIV showed the most consistent association across endpoints after adjustment for selected clinical covariates, while MLR for PFS and PLR for OS provided additional prognostic information. However, these findings should be interpreted as exploratory, given the retrospective design, internally derived cut-offs, and absence of external validation. Prospectively, further research should address this study limitations by: validating the observed effects of PIV, NLR, PLR, MLR and LMR in independent cohorts using pre-specified cut-offs; evaluating on-treatment dynamics of these biomarkers (e.g., during the first two cycles) and their predictive value; integrating inflammatory biomarkers with LDH, ECOG performance status, sites of disease (particularly liver/CNS), and molecular features into multivariable nomograms for prediction of treatment response; testing whether high-risk inflammatory profiles identify patients more likely to benefit from intensified combination regimens versus monotherapy; and combining inflammatory biomarkers with circulating tumor DNA and cell-free DNA in multivariable nomograms to improve predictive performance.

Several limitations should be acknowledged. First, treatment heterogeneity represents an important limitation. Although most patients received anti-PD-1 monotherapy, a small proportion received combination nivolumab/ipilimumab. Second, because of the limited number of patients in the combination subgroup, treatment-specific stratified Cox models or formal interaction testing were not statistically robust. Third, although several clinically relevant prognostic variables were considered, detailed quantitative measures of tumor burden, such as total tumor volume or sum of target lesion diameters, were not consistently available across centers and could not be included in the multivariable models. Fourth, potential multicollinearity among overlapping inflammatory indices may also limit the robustness of individual biomarker-specific associations. Fifth, the ROC-derived biomarker cut-offs were based on one-year overall survival within the same cohort and were not externally or internally validated by bootstrap analysis; therefore, these thresholds should be considered exploratory and require validation in independent prospective cohorts. Finally, findings related to rare histological subtypes, particularly lentigo maligna melanoma, should be interpreted with extreme caution because of the very small number of patients in these subgroups.

## 5. Conclusions

Baseline inflammatory biomarkers derived from routine blood counts were associated with survival outcomes in this real-world cohort of patients with advanced melanoma receiving first-line ICI therapy. Among the evaluated indices, PIV showed the most consistent association after adjustment for selected clinical covariates, suggesting that it may contribute to baseline risk stratification together with established clinical factors such as LDH, ECOG performance status, and metastatic pattern. However, these findings should be considered exploratory because of the retrospective design, internally derived cut-offs, and lack of external validation. Prospective studies using pre-specified thresholds and independent cohorts are needed before these biomarkers can be implemented in routine clinical practice.

## Figures and Tables

**Table 1:** Baseline clinicopathologic characteristics of the cohort.

Characteristic	*N* (%)	Characteristic	*N* (%)
**DEMOGRAPHICS**		**Lymphovascular invasion**	
**Gender**		Present	29 (17.9)
Male	101 (62.35)	Absent	66 (40.44)
Female	61 (37.65)	Unknown/missing	67 (41.36)
**Age at the start of immunotherapy**		**Perineural invasion**	
≤60 years	53 (32.72)	Present	8 (4.94)
>60 years	109 (67.28)	Absent	74 (45.68)
**HISTOPATHOLOGY**		Unknown/missing	80 (49.38)
**Melanoma subtype**		**BRAF mutation status**	
Superficial spreading melanoma	39 (24.07)	Positive	63 (38.89)
Nodular melanoma	89 (54.94)	Wild type	97 (59.88)
Lentigo maligna melanoma	2 (1.23)	Unknown	2 (1.93)
Acral lentiginous melanoma	10 (6.17)	**CLINICAL CHARACTERISTICS**	
Others	22 (13.58)	**Initial clinical stage**	
**Breslow thickness**		I	11 (6.79)
≤4 mm	63 (38.89)	II	90 (55.56)
>4 mm	81 (50)	III	46 (28.4)
Unknown/missing	18 (11.11)	IV	12 (7.41)
**Clark level**		Unknown	3 (1.85)
II	5 (3.09)	**Clinical stage at the start therapy**	
III	25 (15.43)	III	28 (17.28)
IV	81 (50)	IV	134 (82.72)
V	24 (14.81)	**Metastatic sites**	
Not specified	18 (11.11)	Skin/subcutaneous	51 (31.48)
Unknown/missing	9 (5.56)	Lymph node	90 (55.56)
**Ulceration**		Lungs	82 (50.62)
Present	108 (66.67)	Liver	44 (27.16)
Absent	31 (19.14)	Bones	17 (10.49)
Unknown/missing	23 (14.20)	Brain/CNS	17 (10.49)
**Mitotic rate**		Adrenal gland	18 (11.11)
≤1	6 (3.70)	Other	35 (21.6)
>1–2	10 (6.17)	**LDH level**	
>2–4	23 (14.2)	Elevated	31 (19.14)
>4	64 (39.51)	>2× elevated	26 (16.05)
Unknown/missing	59 (36.42)	Normal	100 (61.73)
**Tumor-infiltrating lymphocytes**		Unknown/missing	5 (3.09)
Present	74 (45.68)	**S-100 level**	
Absent	14 (8.64)	Elevated	16 (9.88)
Unknown/missing	74 (45.68)	Normal	20 (12.35)
**Microsatellites**		Unknown/missing	126 (77.78)
Absent	156 (96.30)	**ECOG performance status**	
Present	6 (3.7)	0	104 (64.2)
		1	48 (29.63)
		2	2 (1.23)
		3	1 (0.62)
		Unknown/missing	7 (4.32)

Abbreviations: CNS, central nervous system; ECOG, Eastern Cooperative Oncology Group; LDH, lactate dehydrogenase.

**Table 2:** Association between inflammatory biomarkers and clinical characteristics.

The presence of microsatellites			
	↑ (%)	↓ (%)	*p*
NLR (cut-off 3.748)	8.7	1.72	0.055
PLR (cut-off 180.741)	8.33	1.77	0.065
Elevated serum LDH			
	↑ (%)	↓ (%)	*p*
NLR (cut-off 3.748)	54.35	27.59	0.001
PLR (cut-off 180.741)	58.34	25.66	<0.0001
MLR (cut-off 0.298)	46.63	25.27	0.001
At least one (NLR, PLR or MLR) ↑	46.99	22.79	0.0001
LMR (cut-off 3.351)	25.84	45.32	0.002
S II (cut-off 763.958)	52.46	25	0.0002
PIV (cut-off 277.269)	44.45	24.69	<0.0001
Elevated serum S-100			
	↑ (%)	↓ (%)	*p*
MLR (cut-off 0.298)	20.63	3.3	0.002
S II (cut-off 763.958)	16.39	5	0.051
PIV (cut-off 277.269)	16.67	3.7	0.01
The presence of liver metastasis			
	↑ (%)	↓ (%)	*p*
NLR (cut-off 3.748)	45.65	19.83	0.002
PLR (cut-off 180.741)	43.75	20.35	0.004
MLR (cut-off 0.298)	36.51	20.88	0.043
At least one (NLR, PLR or MLR) ↑	34.94	19.99	0.033
LMR (cut-off 3.351)	21.35	35.94	0.066
S II (cut-off 763.958)	40.98	19	0.003
The presence of more than two metastatic sites			
	↑ (%)	↓ (%)	*p*
PLR (cut-off 180.741)	41.67	24.78	0.039
S II (cut-off 763.958)	39.34	24	0.053
Unfavorable ECOG score			
	↑ (%)	↓ (%)	*p*
NLR (cut-off 3.748)	54.35	22.41	0.001
PLR (cut-off 180.741)	50	23.89	0.004
MLR (cut-off 0.298)	41.27	24.18	0.004
At least one (NLR, PLR or MLR) ↑	43.37	18.99	0.003
LMR (cut-off 3.351)	24.72	40.63	0.051
S II (cut-off 763.958)	45.9	23	0.002
PIV (cut-off 277.269)	41.67	22.22	0.022
Age at the start of immunotherapy			
	↑	↓	*p*
NLR (cut-off 3.748)	70	66	0.043
PLR (cut-off 180.741)	70	65	0.015
MLR (cut-off 0.298)	71	63	0.002
At least one (NLR, PLR or MLR) ↑	70	63	0.005
LMR (cut-off 3.351)	64	70.5	0.004
S II (cut-off 763.958)	69	65	0.031
Poor ORR to therapy			
	↑ (%)	↓ (%)	*p*
NLR (cut-off 3.748)	19.57	43.97	0.004
PLR (cut-off 180.741)	12.5	46.9	<0.0001
MLR (cut-off 0.298)	25.4	42.86	0.028
At least one (NLR, PLR or MLR) ↑	73.49	51.9	0.006
LMR (cut-off 3.351)	25	42.7	0.027
S II (cut-off 763.958)	77.05	55	0.007
PIV (cut-off 277.269)	73.61	56.79	0.042

The table includes significant associations between inflammatory biomarkers and clinical characteristics (*p* < 0.05) and borderline significance (*p* = 0.051 to *p* = 0.066); ↑ (%) the percentage of patients with inflammatory biomarker values above the cut-off; ↓ (%) the percentage of patients with inflammatory biomarker values below the cut-off.

**Table 3:** Significant findings from biomarker-specific multivariable Cox regression models for PFS and OS.

Endpoint	Model	Variable	Comparison/Scale	HR	95% CI	*p*-Value
PFS	NLR	Subtype: ALM	vs. SSM	2.360	1.027–5.423	0.0432
		LDH > 2× elevated	vs. normal	2.299	1.355–3.902	0.0020
		ECOG > 0	vs. 0	1.858	1.141–3.024	0.0127
	PLR	LDH > 2× elevated	vs. normal	2.132	1.191–3.816	0.0108
		ECOG > 0	vs. 0	1.797	1.099–2.940	0.0196
	LMR	Subtype: ALM	vs. SSM	2.590	1.101–6.095	0.0292
		LDH > 2× elevated	vs. normal	1.867	1.072–3.251	0.0273
		ECOG > 0	vs. 0	1.896	1.158–3.103	0.0110
	MLR	MLR	continuous variable, per unit increase	3.323	1.412–7.819	0.0060
		Subtype: ALM	vs. SSM	2.751	1.172–6.456	0.0201
		ECOG > 0	vs. 0	1.846	1.124–3.033	0.0155
	PIV	PIV	continuous variable, per unit increase	1.001	1.000–1.001	0.0013
		Subtype: ALM	vs. SSM	3.092	1.309–7.301	0.0100
	SII	SII	continuous variable, per unit increase	1.000	1.000–1.001	0.0057
		Subtype: ALM	vs. SSM	2.634	1.133–6.124	0.0245
		LDH > 2× elevated	vs. normal	1.870	1.061–3.298	0.0305
		ECOG > 0	vs. 0	1.787	1.092–2.924	0.0208
OS	NLR	Age	continuous variable, per year	1.026	1.003–1.048	0.0250
		Subtype: ALM	vs. SSM	3.835	1.382–10.645	0.0098
		PNI absent	vs. present	0.302	0.113–0.805	0.0167
		LDH elevated	vs. normal	2.466	1.257–4.838	0.0086
		LDH > 2× elevated	vs. normal	4.475	2.216–9.034	<0.0001
	PLR	PLR	continuous variable, per unit increase	1.003	1.000–1.005	0.0316
		Age	continuous variable, per year	1.030	1.007–1.053	0.0089
		Subtype: ALM	vs. SSM	3.253	1.176–9.001	0.0231
		PNI absent	vs. present	0.291	0.108–0.787	0.0149
		LDH elevated	vs. normal	2.224	1.133–4.365	0.0202
		LDH > 2× elevated	vs. normal	3.731	1.779–7.824	0.0005
		CNS metastasis absent	vs. present	0.449	0.209–0.961	0.0392
	LMR	Age	continuous variable, per year	1.029	1.006–1.052	0.0133
		Subtype: ALM	vs. SSM	3.944	1.324–11.746	0.0137
		PNI absent	vs. present	0.327	0.122–0.879	0.0268
		LDH elevated	vs. normal	2.068	1.011–4.231	0.0465
		LDH > 2× elevated	vs. normal	3.217	1.526–6.779	0.0021
		CNS metastasis absent	vs. present	0.441	0.204–0.954	0.0375
	MLR	Age	continuous variable, per year	1.030	1.007–1.053	0.0097
		Subtype: ALM	vs. SSM	4.124	1.397–12.169	0.0103
		PNI absent	vs. present	0.302	0.113–0.810	0.0173
		LDH elevated	vs. normal	2.033	1.002–4.125	0.0493
		LDH > 2× elevated	vs. normal	3.210	1.459–7.063	0.0038
		CNS metastasis absent	vs. present	0.412	0.191–0.891	0.0243
	PIV	PIV	continuous variable, per unit increase	1.000	1.000–1.001	0.0322
		Age	continuous variable, per year	1.037	1.014–1.062	0.0020
		Subtype: ALM	vs. SSM	3.964	1.333–11.788	0.0133
		LDH elevated	vs. normal	2.134	1.052–4.330	0.0357
		LDH > 2× elevated	vs. normal	2.628	1.181–5.846	0.0179
		CNS metastasis absent	vs. present	0.379	0.177–0.811	0.0124
	SII	SII	continuous variable, per unit increase	1.001	1.000–1.001	0.0003
		Age	continuous variable, per year	1.030	1.006–1.054	0.0121
		Subtype: ALM	vs. SSM	4.056	1.440–11.419	0.0080
		LDH elevated	vs. normal	2.159	1.088–4.284	0.0278
		LDH > 2× elevated	vs. normal	3.248	1.532–6.887	0.0021
		CNS metastasis absent	vs. present	0.443	0.205–0.955	0.0378

Only statistically significant, clinically interpretable rows (*p* < 0.05) are shown. Unknown/missing categories are not displayed. Full biomarker-specific multivariable models are provided in Supplementary Tables S2. ALM, acral lentiginous melanoma; SSM, superficial spreading melanoma; PNI, perineural invasion; PFS, progression-free survival; OS, overall survival; HR, hazard ratio; CI, confidence interval; NLR, neutrophil-to-lymphocyte ratio; PLR, platelet-to-lymphocyte ratio; LMR, lymphocyte-to-monocyte ratio; MLR, monocyte-to-lymphocyte ratio; PIV, pan-immune-inflammation value; SII, systemic immune-inflammation index; LDH, lactate dehydrogenase; ECOG, Eastern Cooperative Oncology Group; CNS, central nervous system.

## Data Availability

The data underlying this article will be shared on reasonable request to the corresponding author.

## Supplementary Materials

The following supporting information can be downloaded at: https://www.mdpi.com/article/10.3390/cancers18111722/s1, Table S1: Uniivariable Cox regression models for progression-free survival (PFS) and overall survival (OS) (biomarker-specific models adjusted for baseline clinical covariates); Table S2: Multivariable Cox regression models for progression-free survival (PFS) overall survival (OS) (biomarker-specific models adjusted for baseline clinical covariates).
