# Supplementary material for: Prognostic Significance of Inflammatory Biomarkers in First-Line Immunotherapy for Metastatic Melanoma: Multicentric Study
Source: Cancers (Basel). 2026 May 25;18(11):1722. doi: 10.3390/cancers18111722 (PMC13255854; doi:10.3390/cancers18111722)
Supplement: Supplementary file 1 [file cancers-18-01722-s001.zip › cancers-4301393-supplementary.pdf]

Supplementary Table S1. Multivariable Cox regression models for progression-free survival (PFS) (biomarker-specific models adjusted for baseline clinical covariates).

PFS- Multivariate Cox-Regression- Model with NLR

Variable	*Value*	*N*	*with Event*	*Censored*	*Estimate (Std)*	*HR (CI95)*	*p-value*	*p-value (effect)*
NLR	Continuous variable	162	79	83	0.058 (0.035)	1.060 (0.990- 1.134)	0.0956	0.0956
Age	Continuous variable	162	79	83	0.005 (0.008)	1.005 (0.990- 1.021)	0.5088	0.5088
Subtype	Superficial spreading melanoma	39	12	27	Reference			0.0569
Subtype	Nodular melanoma	89	50	39	-0.037 (0.260)	0.963 (0.579- 1.603)	0.8857	
Subtype	Lentigo maligna melanoma	2	0	2	-13.179 (560.489)	0.000 (0.000)	0.9812	
Subtype	Acral lentiginous melanoma	10	8	2	0.859 (0.425)	2.360 (1.027- 5.423)	0.0432	
Subtype	Others	22	9	13	-0.503 (0.348)	0.605 (0.306- 1.196)	0.1482	
Perineural invasion	Present	8	6	2	Reference			0.1257
Perineural invasion	Absent	74	33	41	-0.847 (0.438)	0.429 (0.182- 1.012)	0.0531	
Perineural invasion	Unknown/Missing	80	40	40	-0.623 (0.441)	0.536 (0.226- 1.273)	0.1578	
LDH	Normal	100	35	65	Reference			0.0072
LDH	Elevated	31	20	11	0.232 (0.275)	1.261 (0.736- 2.161)	0.3982	
LDH	>2xElevated	26	21	5	0.833 (0.270)	2.299 (1.355- 3.902)	0.0020	
LDH	Missing	5	3	2	1.040 (0.534)	2.828 (0.994- 8.048)	0.0514	
ECOG	0	104	39	65	Reference			0.0246
ECOG	>0	51	36	15	0.619 (0.249)	1.858 (1.141- 3.024)	0.0127	
ECOG	Unknown/Missing	7	4	3	-0.419 (0.672)	0.658 (0.176- 2.453)	0.5327	

PFS- Multivariate Cox-Regression- Model with PLR

Variable	*Value*	*N*	*with Event*	*Censored*	*Estimate (Std)*	*HR (CI95)*	*p-value*	*p-value (effect)*
PLR	Continuous variable	161	79	82	0.001 (0.001)	1.001 (0.999- 1.004)	0.2808	0.2808
Age	Continuous variable	161	79	82	0.009 (0.008)	1.009 (0.994- 1.025)	0.2507	0.2507
Subtype	Superficial spreading melanoma	39	12	27	Reference			0.0487
Subtype	Nodular melanoma	88	50	38	0.058 (0.259)	1.060 (0.638- 1.762)	0.8222	
Subtype	Lentigo maligna melanoma	2	0	2	-13.054 (554.998)	0.000 (0.000)	0.9812	
Subtype	Acral lentiginous melanoma	10	8	2	0.809 (0.424)	2.245 (0.978- 5.155)	0.0565	
Subtype	Others	22	9	13	-0.562 (0.349)	0.570 (0.288- 1.130)	0.1074	
Perineural invasion	Present	8	6	2	Reference			0.1219
Perineural invasion	Absent	74	33	41	-0.842 (0.442)	0.431 (0.181- 1.024)	0.0566	
Perineural invasion	Unknown/Missing	79	40	39	-0.595 (0.445)	0.551 (0.231- 1.318)	0.1804	
LDH	Normal	99	35	64	Reference			0.0321
LDH	Elevated	31	20	11	0.177 (0.278)	1.194 (0.693- 2.057)	0.5235	
LDH	>2xElevated	26	21	5	0.757 (0.297)	2.132 (1.191- 3.816)	0.0108	
LDH	Missing	5	3	2	0.933 (0.539)	2.541 (0.884- 7.308)	0.0836	
ECOG	0	104	39	65	Reference			0.0620
ECOG	>0	51	36	15	0.586 (0.251)	1.797 (1.099- 2.940)	0.0196	
ECOG	Unknown/Missing	6	4	2	0.398 (0.540)	1.488 (0.516- 4.291)	0.4617	

PFS- Multivariate Cox-Regression- Model with LMR

Variable	*Value*	*N*	*with Event*	*Censored*	*Estimate (Std)*	*HR (CI95)*	*p-value*	*p-value (effect)*
LMR	Continuous variable	153	75	78	-0.080 (0.052)	0.923 (0.833- 1.022)	0.1237	0.1237
Age	Continuous variable	153	75	78	0.008 (0.007)	1.008 (0.993- 1.023)	0.3132	0.3132
Subtype	Superficial spreading melanoma	36	9	27	Reference			0.2016
Subtype	Nodular melanoma	87	50	37	0.144 (0.271)	1.155 (0.679- 1.965)	0.5949	
Subtype	Lentigo maligna melanoma	2	0	2	-12.883 (539.734)	0.000 (0.000)	0.9810	
Subtype	Acral lentiginous melanoma	10	8	2	0.952 (0.437)	2.590 (1.101- 6.095)	0.0292	
Subtype	Others	18	8	10	-0.067 (0.368)	0.935 (0.455- 1.924)	0.8559	
Perineural invasion	Present	8	6	2	Reference			0.2333
Perineural invasion	Absent	71	32	39	-0.714 (0.442)	0.490 (0.206- 1.165)	0.1065	
Perineural invasion	Unknown/Missing	74	37	37	-0.510 (0.444)	0.600 (0.252- 1.432)	0.2499	
LDH	Normal	96	35	61	Reference			0.0458
LDH	Elevated	27	17	10	0.166 (0.296)	1.181 (0.662- 2.107)	0.5741	
LDH	>2xElevated	25	20	5	0.624 (0.283)	1.867 (1.072- 3.251)	0.0273	
LDH	Missing	5	3	2	1.072 (0.546)	2.920 (1.002- 8.511)	0.0496	
ECOG	0	100	38	62	Reference			0.0332
ECOG	>0	48	35	13	0.640 (0.251)	1.896 (1.158- 3.103)	0.0110	
ECOG	Unknown/Missing	5	2	3	-0.092 (0.626)	0.912 (0.267- 3.113)	0.8830	

PFS- Multivariate Cox-Regression- Model with MLR

Variable	*Value*	*N*	*with Event*	*Censored*	*Estimate (Std)*	*HR (CI95)*	*p-value*	*p-value (effect)*
MLR	Continuous variable	154	75	79	1.201 (0.437)	3.323 (1.412- 7.819)	0.0060	0.0060
Age	Continuous variable	154	75	79	0.005 (0.008)	1.005 (0.990- 1.021)	0.4786	0.4786
Subtype	Superficial spreading melanoma	36	9	27	Reference			0.1029
Subtype	Nodular melanoma	87	50	37	0.123 (0.271)	1.131 (0.665- 1.923)	0.6491	
Subtype	Lentigo maligna melanoma	2	0	2	-13.015 (559.815)	0.000 (0.000)	0.9815	
Subtype	Acral lentiginous melanoma	10	8	2	1.012 (0.435)	2.751 (1.172- 6.456)	0.0201	
Subtype	Others	19	8	11	-0.203 (0.373)	0.816 (0.393- 1.695)	0.5860	
Perineural invasion	Present	8	6	2	Reference			0.1505
Perineural invasion	Absent	71	32	39	-0.814 (0.444)	0.443 (0.186- 1.057)	0.0665	
Perineural invasion	Unknown/Missing	75	37	38	-0.577 (0.445)	0.561 (0.235- 1.343)	0.1946	
LDH	Normal	97	35	62	Reference			0.1372
LDH	Elevated	27	17	10	0.138 (0.291)	1.148 (0.649- 2.032)	0.6348	
LDH	>2xElevated	25	20	5	0.473 (0.302)	1.604 (0.887- 2.900)	0.1178	
LDH	Missing	5	3	2	1.025 (0.538)	2.788 (0.972- 7.995)	0.0565	
ECOG	0	101	38	63	Reference			0.0389
ECOG	>0	48	35	13	0.613 (0.253)	1.846 (1.124- 3.033)	0.0155	
ECOG	Unknown/Missing	5	2	3	-0.232 (0.633)	0.793 (0.229- 2.741)	0.7137	

PFS- Multivariate Cox-Regression- Model with PIV

Variable	*Value*	*N*	*with Event*	*Censored*	*Estimate (Std)*	*HR (CI95)*	*p-value*	*p-value (effect)*
PIV	Continuous variable	153	75	78	0.001 (0.000)	1.001 (1.000- 1.001)	0.0013	0.0013
Age	Continuous variable	153	75	78	0.011 (0.008)	1.011 (0.995- 1.027)	0.1641	0.1641
Subtype	Superficial spreading melanoma	36	9	27	Reference			0.0724
Subtype	Nodular melanoma	86	50	36	0.192 (0.277)	1.211 (0.704- 2.083)	0.4886	
Subtype	Lentigo maligna melanoma	2	0	2	-13.059 (606.607)	0.000 (0.000)	0.9828	
Subtype	Acral lentiginous melanoma	10	8	2	1.129 (0.438)	3.092 (1.309- 7.301)	0.0100	
Subtype	Others	19	8	11	-0.112 (0.370)	0.894 (0.433- 1.846)	0.7613	
Perineural invasion	Present	8	6	2	Reference			0.4800
Perineural invasion	Absent	71	32	39	-0.086 (0.520)	0.918 (0.331- 2.543)	0.8690	
Perineural invasion	Unknown/Missing	74	37	37	0.182 (0.536)	1.199 (0.419- 3.431)	0.7349	
LDH	Normal	96	35	61	Reference			0.4456
LDH	Elevated	27	17	10	0.203 (0.291)	1.224 (0.692- 2.168)	0.4871	
LDH	>2xElevated	25	20	5	0.412 (0.307)	1.510 (0.828- 2.756)	0.1791	
LDH	Missing	5	3	2	0.639 (0.558)	1.894 (0.635- 5.651)	0.2522	
ECOG	0	101	38	63	Reference			0.1140
ECOG	>0	48	35	13	0.512 (0.262)	1.668 (0.998- 2.788)	0.0510	
ECOG	Unknown/Missing	4	2	2	0.639 (0.748)	1.894 (0.437- 8.212)	0.3935	

PFS- Multivariate Cox-Regression- Model with SII

Variable	*Value*	*N*	*with Event*	*Censored*	*Estimate (Std)*	*HR (CI95)*	*p-value*	*p-value (effect)*
SII	Continuous variable	161	79	82	0.000 (0.000)	1.000 (1.000- 1.001)	0.0057	0.0057
Age	Continuous variable	161	79	82	0.009 (0.008)	1.009 (0.993- 1.024)	0.2788	0.2788
Subtype	Superficial spreading melanoma	39	12	27	Reference			0.0292
Subtype	Nodular melanoma	88	50	38	0.058 (0.261)	1.060 (0.636- 1.768)	0.8233	
Subtype	Lentigo maligna melanoma	2	0	2	-13.029 (577.737)	0.000 (0.000)	0.9820	
Subtype	Acral lentiginous melanoma	10	8	2	0.968 (0.431)	2.634 (1.133- 6.124)	0.0245	
Subtype	Others	22	9	13	-0.518 (0.353)	0.595 (0.298- 1.189)	0.1416	
Perineural invasion	Present	8	6	2	Reference			0.3207
Perineural invasion	Absent	74	33	41	-0.391 (0.493)	0.676 (0.257- 1.777)	0.4273	
Perineural invasion	Unknown/Missing	79	40	39	-0.098 (0.506)	0.907 (0.336- 2.444)	0.8464	
LDH	Normal	99	35	64	Reference			0.1131
LDH	Elevated	31	20	11	0.157 (0.278)	1.171 (0.679- 2.018)	0.5709	
LDH	>2xElevated	26	21	5	0.626 (0.289)	1.870 (1.061- 3.298)	0.0305	
LDH	Missing	5	3	2	0.807 (0.543)	2.240 (0.773- 6.489)	0.1372	
ECOG	0	104	39	65	Reference			0.0596
ECOG	>0	51	36	15	0.581 (0.251)	1.787 (1.092- 2.924)	0.0208	
ECOG	Unknown/Missing	6	4	2	0.538 (0.547)	1.713 (0.586- 5.006)	0.3250	

PFS, progression-free survival; HR, hazard ratio; CI, confidence interval; NLR, neutrophil-to-lymphocyte ratio; PLR, platelet-to-lymphocyte ratio; MLR, monocyte-to-lymphocyte ratio; LMR, lymphocyte-to-monocyte ratio; SII, systemic immune-inflammation index; PIV, pan-immune-inflammation value.

Supplementary Table S2. Multivariable Cox regression models for overall survival (OS) (biomarker-specific models adjusted for baseline clinical covariates).

OS- Multivariate Cox-Regression- Model with NLR

Variable	*Value*	*N*	*with Event*	*Censored*	*Estimate (Std)*	*HR (CI95)*	*p-value*	*p-value (effect)*
NLR	Continuous variable	162	79	83	0.038 (0.040)	1.039 (0.960- 1.124)	0.3448	0.3448
Age	Continuous variable	162	79	83	0.025 (0.011)	1.026 (1.003- 1.048)	0.0250	0.0250

Variable	*Value*	*N*	*with Event*	*Censored*	*Estimate (Std)*	*HR (CI95)*	*p-value*	*p-value (effect)*
Subtype	Superficial spreading melanoma	39	12	27	Reference			0.0154
Subtype	Nodular melanoma	89	50	39	0.365 (0.347)	1.441 (0.730- 2.846)	0.2925	
Subtype	Lentigo maligna melanoma	2	0	2	-13.219 (766.859)	0.000 (0.000)	0.9862	
Subtype	Acral lentiginous melanoma	10	8	2	1.344 (0.521)	3.835 (1.382- 10.645)	0.0098	
Subtype	Others	22	9	13	-0.474 (0.468)	0.623 (0.249- 1.559)	0.3117	
Perineural invasion	Present	8	6	2	Reference			0.0566
Perineural invasion	Absent	74	33	41	-1.197 (0.500)	0.302 (0.113- 0.805)	0.0167	
Perineural invasion	Unknown/Missing	80	40	40	-1.086 (0.511)	0.337 (0.124- 0.918)	0.0334	
BRAF	Positive	63	23	40	Reference			0.4150
BRAF	Unknown	2	1	1	0.998 (1.400)	2.714 (0.175- 42.187)	0.4757	
BRAF	Wild-type	97	55	42	-0.306 (0.307)	0.736 (0.403- 1.343)	0.3182	
Metastatic sites	1	52	22	30	Reference			0.7832
Metastatic sites	>=2	110	57	53	-0.086 (0.313)	0.917 (0.496- 1.695)	0.7832	
LDH	Normal	100	35	65	Reference			0.0003
LDH	Elevated	31	20	11	0.903 (0.344)	2.466 (1.257- 4.838)	0.0086	
LDH	>2xElevated	26	21	5	1.498 (0.358)	4.475 (2.216- 9.034)	<.0001	
LDH	Missing	5	3	2	0.909 (0.753)	2.483 (0.567- 10.865)	0.2272	
CNS Metastasis	Yes	17	11	6	Reference			0.0556
CNS Metastasis	No	145	68	77	-0.756 (0.395)	0.470 (0.217- 1.018)	0.0556	
Liver Metastasis	Yes	44	26	18	Reference			0.9177
Liver Metastasis	No	118	53	65	0.031 (0.298)	1.031 (0.576- 1.848)	0.9177	
ECOG	0	104	39	65	Reference			0.1882
ECOG	>0	51	36	15	0.570 (0.313)	1.768 (0.957- 3.266)	0.0687	
ECOG	Unknown/Missing	7	4	3	0.124 (0.717)	1.132 (0.278- 4.615)	0.8631	

OS- Multivariate Cox-Regression- Model with PLR

Variable	*Value*	*N*	*with Event*	*Censored*	*Estimate (Std)*	*HR (CI95)*	*p-value*	*p-value (effect)*
PLR	Continuous variable	161	79	82	0.003 (0.001)	1.003 (1.000- 1.005)	0.0316	0.0316
Age	Continuous variable	161	79	82	0.030 (0.011)	1.030 (1.007- 1.053)	0.0089	0.0089
Subtype	Superficial spreading melanoma	39	12	27	Reference			0.0105
Subtype	Nodular melanoma	88	50	38	0.476 (0.345)	1.609 (0.819- 3.162)	0.1678	
Subtype	Lentigo maligna melanoma	2	0	2	-13.191 (766.501)	0.000 (0.000)	0.9863	
Subtype	Acral lentiginous melanoma	10	8	2	1.180 (0.519)	3.253 (1.176- 9.001)	0.0231	
Subtype	Others	22	9	13	-0.622 (0.475)	0.537 (0.211- 1.362)	0.1905	
Perineural invasion	Present	8	6	2	Reference			0.0517
Perineural invasion	Absent	74	33	41	-1.233 (0.507)	0.291 (0.108- 0.787)	0.0149	
Perineural invasion	Unknown/Missing	79	40	39	-1.096 (0.517)	0.334 (0.121- 0.920)	0.0339	
BRAF	Positive	63	23	40	Reference			0.3849
BRAF	Unknown	2	1	1	1.443 (1.367)	4.232 (0.291- 61.641)	0.2911	
BRAF	Wild-type	96	55	41	-0.226 (0.307)	0.798 (0.437- 1.455)	0.4611	
Metastatic sites	1	52	22	30	Reference			0.8230
Metastatic sites	>=2	109	57	52	0.069 (0.309)	1.072 (0.585- 1.964)	0.8230	
LDH	Normal	99	35	64	Reference			0.0050
LDH	Elevated	31	20	11	0.799 (0.344)	2.224 (1.133- 4.365)	0.0202	
LDH	>2xElevated	26	21	5	1.317 (0.378)	3.731 (1.779- 7.824)	0.0005	
LDH	Missing	5	3	2	0.630 (0.776)	1.877 (0.410- 8.587)	0.4171	
CNS Metastasis	Yes	17	11	6	Reference			0.0392
CNS Metastasis	No	144	68	76	-0.802 (0.389)	0.449 (0.209- 0.961)	0.0392	
Liver Metastasis	Yes	44	26	18	Reference			0.3279
Liver Metastasis	No	117	53	64	0.308 (0.315)	1.361 (0.734- 2.525)	0.3279	
ECOG	0	104	39	65	Reference			0.0555
ECOG	>0	51	36	15	0.541 (0.312)	1.717 (0.931- 3.168)	0.0836	
ECOG	Unknown/Missing	6	4	2	1.186 (0.592)	3.273 (1.026- 10.443)	0.0452	

OS- Multivariate Cox-Regression- Model with LMR

Variable	*Value*	*N*	*with Event*	*Censored*	*Estimate (Std)*	*HR (CI95)*	*p-value*	*p-value (effect)*
LMR	Continuous variable	153	75	78	-0.073 (0.069)	0.930 (0.812- 1.065)	0.2941	0.2941
Age	Continuous variable	153	75	78	0.028 (0.011)	1.029 (1.006- 1.052)	0.0133	0.0133
Subtype	Superficial spreading melanoma	36	9	27	Reference			0.0693
Subtype	Nodular melanoma	87	50	37	0.651 (0.387)	1.917 (0.899- 4.090)	0.0922	
Subtype	Lentigo maligna melanoma	2	0	2	-12.639 (735.504)	0.000 (0.000)	0.9863	
Subtype	Acral lentiginous melanoma	10	8	2	1.372 (0.557)	3.944 (1.324- 11.746)	0.0137	
Subtype	Others	18	8	10	0.055 (0.514)	1.056 (0.386- 2.892)	0.9152	
Perineural invasion	Present	8	6	2	Reference			0.0858
Perineural invasion	Absent	71	32	39	-1.117 (0.505)	0.327 (0.122- 0.879)	0.0268	
Perineural invasion	Unknown/Missing	74	37	37	-0.954 (0.514)	0.385 (0.141- 1.056)	0.0638	
BRAF	Positive	62	23	39	Reference			0.7163
BRAF	Unknown	2	1	1	0.997 (1.370)	2.711 (0.185- 39.752)	0.4666	
BRAF	Wild-type	89	51	38	-0.097 (0.330)	0.907 (0.475- 1.731)	0.7676	
Metastatic sites	1	48	20	28	Reference			0.6893
Metastatic sites	>=2	105	55	50	0.129 (0.322)	1.137 (0.605- 2.138)	0.6893	
LDH	Normal	96	35	61	Reference			0.0102
LDH	Elevated	27	17	10	0.727 (0.365)	2.068 (1.011- 4.231)	0.0465	
LDH	>2xElevated	25	20	5	1.168 (0.380)	3.217 (1.526- 6.779)	0.0021	
LDH	Missing	5	3	2	0.926 (0.758)	2.525 (0.572- 11.153)	0.2217	
CNS Metastasis	Yes	16	11	5	Reference			0.0375
CNS Metastasis	No	137	64	73	-0.818 (0.393)	0.441 (0.204- 0.954)	0.0375	
Liver Metastasis	Yes	42	24	18	Reference			0.4333
Liver Metastasis	No	111	51	60	0.251 (0.320)	1.285 (0.686- 2.406)	0.4333	
ECOG	0	100	38	62	Reference			0.2431
ECOG	>0	48	35	13	0.522 (0.322)	1.685 (0.897- 3.165)	0.1046	
ECOG	Unknown/Missing	5	2	3	-0.204 (0.803)	0.815 (0.169- 3.935)	0.7993	

OS- Multivariate Cox-Regression- Model with MLR

Variable	*Value*	*N*	*with Event*	*Censored*	*Estimate (Std)*	*HR (CI95)*	*p-value*	*p-value (effect)*
MLR	Continuous variable	154	75	79	0.485 (0.544)	1.624 (0.559- 4.718)	0.3732	0.3732
Age	Continuous variable	154	75	79	0.029 (0.011)	1.030 (1.007- 1.053)	0.0097	0.0097
Subtype	Superficial spreading melanoma	36	9	27	Reference			0.0484
Subtype	Nodular melanoma	87	50	37	0.654 (0.387)	1.923 (0.901- 4.106)	0.0909	
Subtype	Lentigo maligna melanoma	2	0	2	-12.716 (759.599)	0.000 (0.000)	0.9866	
Subtype	Acral lentiginous melanoma	10	8	2	1.417 (0.552)	4.124 (1.397- 12.169)	0.0103	
Subtype	Others	19	8	11	0.016 (0.513)	1.017 (0.372- 2.781)	0.9744	
Perineural invasion	Present	8	6	2	Reference			0.0589
Perineural invasion	Absent	71	32	39	-1.197 (0.503)	0.302 (0.113- 0.810)	0.0173	
Perineural invasion	Unknown/Missing	75	37	38	-1.018 (0.509)	0.361 (0.133- 0.980)	0.0455	
BRAF	Positive	63	23	40	Reference			0.6704
BRAF	Unknown	2	1	1	1.036 (1.370)	2.818 (0.192- 41.281)	0.4495	
BRAF	Wild-type	89	51	38	-0.127 (0.327)	0.881 (0.464- 1.673)	0.6988	
Metastatic sites	1	48	20	28	Reference			0.7368
Metastatic sites	>=2	106	55	51	0.108 (0.321)	1.114 (0.593- 2.092)	0.7368	
LDH	Normal	97	35	62	Reference			0.0184
LDH	Elevated	27	17	10	0.710 (0.361)	2.033 (1.002- 4.125)	0.0493	
LDH	>2xElevated	25	20	5	1.166 (0.402)	3.210 (1.459- 7.063)	0.0038	
LDH	Missing	5	3	2	0.829 (0.749)	2.291 (0.528- 9.938)	0.2681	
CNS Metastasis	Yes	16	11	5	Reference			0.0243
CNS Metastasis	No	138	64	74	-0.887 (0.394)	0.412 (0.191- 0.891)	0.0243	
Liver Metastasis	Yes	42	24	18	Reference			0.3831
Liver Metastasis	No	112	51	61	0.282 (0.323)	1.325 (0.704- 2.496)	0.3831	
ECOG	0	101	38	63	Reference			0.2949
ECOG	>0	48	35	13	0.490 (0.329)	1.632 (0.857- 3.110)	0.1362	
ECOG	Unknown/Missing	5	2	3	-0.203 (0.810)	0.816 (0.167- 3.996)	0.8022	

OS- Multivariate Cox-Regression- Model with PIV

Variable	*Value*	*N*	*with Event*	*Censored*	*Estimate (Std)*	*HR (CI95)*	*p-value*	*p-value (effect)*
PIV	Continuous variable	153	75	78	0.000 (0.000)	1.000 (1.000- 1.001)	0.0322	0.0322
Age	Continuous variable	153	75	78	0.037 (0.012)	1.037 (1.014- 1.062)	0.0020	0.0020
Subtype	Superficial spreading melanoma	36	9	27	Reference			0.0631
Subtype	Nodular melanoma	86	50	36	0.676 (0.390)	1.967 (0.916- 4.222)	0.0827	
Subtype	Lentigo maligna melanoma	2	0	2	-12.601 (915.565)	0.000 (0.000)	0.9890	
Subtype	Acral lentiginous melanoma	10	8	2	1.377 (0.556)	3.964 (1.333- 11.788)	0.0133	
Subtype	Others	19	8	11	0.052 (0.515)	1.054 (0.384- 2.890)	0.9188	
Perineural invasion	Present	8	6	2	Reference			0.2238
Perineural invasion	Absent	71	32	39	-0.817 (0.553)	0.442 (0.150- 1.305)	0.1395	
Perineural invasion	Unknown/Missing	74	37	37	-0.517 (0.586)	0.596 (0.189- 1.880)	0.3776	
BRAF	Positive	63	23	40	Reference			0.8509
BRAF	Unknown	2	1	1	0.615 (1.435)	1.849 (0.111- 30.804)	0.6685	
BRAF	Wild-type	88	51	37	-0.102 (0.317)	0.903 (0.486- 1.680)	0.7476	
Metastatic sites	1	48	20	28	Reference			0.5244
Metastatic sites	>=2	105	55	50	0.207 (0.325)	1.230 (0.651- 2.324)	0.5244	
LDH	Normal	96	35	61	Reference			0.0608
LDH	Elevated	27	17	10	0.758 (0.361)	2.134 (1.052- 4.330)	0.0357	
LDH	>2xElevated	25	20	5	0.966 (0.408)	2.628 (1.181- 5.846)	0.0179	
LDH	Missing	5	3	2	0.441 (0.786)	1.554 (0.333- 7.250)	0.5744	
CNS Metastasis	Yes	16	11	5	Reference			0.0124
CNS Metastasis	No	137	64	73	-0.970 (0.388)	0.379 (0.177- 0.811)	0.0124	
Liver Metastasis	Yes	42	24	18	Reference			0.2103
Liver Metastasis	No	111	51	60	0.414 (0.330)	1.513 (0.792- 2.891)	0.2103	
ECOG	0	101	38	63	Reference			0.1779
ECOG	>0	48	35	13	0.388 (0.332)	1.475 (0.769- 2.828)	0.2421	
ECOG	Unknown/Missing	4	2	2	1.190 (0.807)	3.288 (0.676- 15.999)	0.1403	

OS- Multivariate Cox-Regression- Model with SII

Variable	*Value*	*N*	*with Event*	*Censored*	*Estimate (Std)*	*HR (CI95)*	*p-value*	*p-value (effect)*
SII	Continuous variable	161	79	82	0.001 (0.000)	1.001 (1.000- 1.001)	0.0003	0.0003
Age	Continuous variable	161	79	82	0.029 (0.012)	1.030 (1.006- 1.054)	0.0121	0.0121
Subtype	Superficial spreading melanoma	39	12	27	Reference			0.0071
Subtype	Nodular melanoma	88	50	38	0.389 (0.352)	1.476 (0.740- 2.944)	0.2689	
Subtype	Lentigo maligna melanoma	2	0	2	-12.775 (926.354)	0.000 (0.000)	0.9890	
Subtype	Acral lentiginous melanoma	10	8	2	1.400 (0.528)	4.056 (1.440- 11.419)	0.0080	
Subtype	Others	22	9	13	-0.605 (0.482)	0.546 (0.212- 1.404)	0.2093	
Perineural invasion	Present	8	6	2	Reference			0.3782
Perineural invasion	Absent	74	33	41	-0.645 (0.556)	0.525 (0.177- 1.559)	0.2459	
Perineural invasion	Unknown/Missing	79	40	39	-0.402 (0.583)	0.669 (0.213- 2.098)	0.4904	
BRAF	Positive	63	23	40	Reference			0.7223
BRAF	Unknown	2	1	1	0.337 (1.402)	1.401 (0.090- 21.872)	0.8099	
BRAF	Wild-type	96	55	41	-0.232 (0.314)	0.793 (0.428- 1.469)	0.4612	
Metastatic sites	1	52	22	30	Reference			0.6840
Metastatic sites	>=2	109	57	52	0.129 (0.317)	1.137 (0.612- 2.115)	0.6840	
LDH	Normal	99	35	64	Reference			0.0179
LDH	Elevated	31	20	11	0.769 (0.350)	2.159 (1.088- 4.284)	0.0278	
LDH	>2xElevated	26	21	5	1.178 (0.383)	3.248 (1.532- 6.887)	0.0021	
LDH	Missing	5	3	2	0.584 (0.782)	1.793 (0.387- 8.305)	0.4552	
CNS Metastasis	Yes	17	11	6	Reference			0.0378
CNS Metastasis	No	144	68	76	-0.814 (0.392)	0.443 (0.205- 0.955)	0.0378	
Liver Metastasis	Yes	44	26	18	Reference			0.2905
Liver Metastasis	No	117	53	64	0.330 (0.312)	1.391 (0.754- 2.565)	0.2905	
ECOG	0	104	39	65	Reference			0.0281
ECOG	>0	51	36	15	0.620 (0.319)	1.859 (0.995- 3.476)	0.0520	
ECOG	Unknown/Missing	6	4	2	1.360 (0.605)	3.898 (1.191- 12.753)	0.0245	

OS, overall survival; HR, hazard ratio; CI, confidence interval; NLR, neutrophil-to-lymphocyte ratio; PLR, platelet-to-lymphocyte ratio; MLR, monocyte-to-lymphocyte ratio; LMR, lymphocyte-to-monocyte ratio; SII, systemic immune-inflammation index; PIV, pan-immune-inflammation value
